# YAP1 mediates the dimensional and chemical coordination of immunoregulation and therapy in extensively passaged mesenchymal stem cells

**DOI:** 10.7150/thno.103314

**Published:** 2025-01-06

**Authors:** Fang-Ying Du, Feng Zhou, Na Zhao, Lei Bao, Cheng-Biao Hu, Jing Lei, An-Qi Liu, Ying-Feng Gao, Li-Hui Bao, Hua Ni, Xiao-Rui Yu, Ji Chen, Bing-Dong Sui

**Affiliations:** 1 Department of Biochemistry and Molecular Biology, School of Basic Medical Sciences, Xi'an Jiaotong University, Xi'an, Shaanxi 710061, China.; 2 State Key Laboratory of Oral & Maxillofacial Reconstruction and Regeneration, National Clinical Research Center for Oral Diseases, Shaanxi International Joint Research Center for Oral Diseases, Center for Tissue Engineering, School of Stomatology, The Fourth Military Medical University, Xi'an, Shaanxi 710032, China.; 3 Xi'an Institute of Tissue Engineering and Regenerative Medicine, Xi'an, Shaanxi 710032, China.; 4 Xi'an Key Laboratory of Stem Cell and Regenerative Medicine, Institute of Medical Research, Northwestern Polytechnical University, Xi'an, Shaanxi 710072, China.; 5 Department of Obstetrics and Gynecology, Xi'an No. 4 Hospital, Affiliated Guangren Hospital, School of Medicine, Xi'an Jiaotong University, Xi'an, Shaanxi 710004, China.; 6 Department of Oral Implantology, School of Stomatology, The Fourth Military Medical University, Xi'an, Shaanxi 710032, China.

**Keywords:** mesenchymal stem cells, immunoregulation, cell therapy, 3D culture, YAP1

## Abstract

**Rationale:** Mesenchymal stem cells (MSCs) possess potent immunomodulatory capability, but occasionally, clinical application of MSCs is hindered by compromised cell functionality and insufficient therapeutic efficacy.

**Methods:** Here, well-established mouse models of dextran sulfate sodium (DSS)-induced colitis and streptozotocin (STZ)-induced type 1 diabetes (T1D) were used to evaluate therapeutic immunomodulatory effects of human umbilical cord-derived MSCs. MSCs were examined at the fifth (P5) and the fifteenth (P15) passages, and three-dimensional (3D) culture was conducted by Matrigel incorporation. A series of biochemical, histopathological and cellular assays were performed to investigate the MSC function and therapeutic performance, and immunoregulation was evaluated by *in vitro* co-culture with T cells and *in vivo* analyses of T-cell infiltration into target tissues. RNA sequencing (RNA-seq) analysis followed by immunofluorescence staining, gene expression analyses and chemical regulation were used to investigate the molecular targets.

**Results:** MSCs lose therapeutic immunomodulatory effects after extensive expansion to P15 when cell senescence occurs. Intriguingly, 3D preconditioning of MSCs in Matrigel promotes diminished immunoregulatory capability despite extensive passages, which benefits function of P15-MSCs to modulate T-cell subsets in co-culture, suppress infiltration of pro-inflammatory T cells in the colon and pancreas tissues after infusion, ameliorate systemic inflammation, and alleviate colitis and T1D in mice. Mechanistically, 3D culture provokes transcriptomic reprogramming of MSCs toward a Yes-associated protein 1 (YAP1)-marked, Hippo signaling pathway-upregulated state with promoted release of the anti-inflammatory cytokine, transforming growth factor-beta1 (TGF-β1). Moreover, chemical regulation of YAP1 by clinically relevant drugs, verteporfin (VP) and prostaglandin E2 (PGE2), affects TGF-β1 expression and the immunomodulatory capability of MSCs during dimensional culture.

**Conclusions:** Taken together, these findings unravel YAP1-based dimensional and chemical coordination of expanded MSC immunoregulation, which will shed light on precisely controlled translational application.

## Introduction

Mesenchymal stem cells (MSCs, also known as mesenchymal stromal cells) exist in almost all tissues/organs as heterogeneous and primitive cells which play putative roles of maintaining tissue/organ homeostasis and contributing to tissue repair and regeneration 1, 2. Notably,* in vitro* expanded MSCs are further employed as therapeutics for autoimmune and inflammatory diseases, including graft-versus-host disease (GVHD), systemic lupus erythematosus (SLE), inflammatory bowel disease (IBD) and type 1 diabetes (T1D), based on the capacity of MSCs to regulate a wide range of immune cells 3, 4. To date, thousands of MSC-based clinical trials for immunotherapy have been registered across many countries, which is becoming one of the most promising and productive fields of MSC research and application 3, 4. However, these trials have achieved inconsistent therapeutic outcomes, and the immunomodulation of MSCs has not been completely fulfilled in clinical settings, which is largely attributed to compromised cell functionality in complicated recipient disease microenvironments 5-9. Thus, convenient approaches to improve the therapeutic performance of MSCs with better understanding of the mechanisms guaranteeing the immunomodulatory efficacy of MSCs are of vital importance to achieve the intended goals of translational application.

The therapeutically used MSCs vary in cell source and production methods, which greatly determine how the infused MSCs function in recipients 10. For a particular example, scalable expansion of MSCs through extensive passages is therapeutically necessary yet subjected to limitations of quality decay, which diminishes stemness, increases cellular senescence and affect the immunomodulatory capacity despite controversial findings 11-18. Intriguingly, three-dimensional (3D) hydrogel environments resembling the physiological tissue microarchitecture benefit functionality of encapsulated MSCs, which retain differentiation and immunomodulation and elevate their *in vivo* efficacy of regeneration and therapy 19-22. It has been documented that local nascent protein deposition and remodelling guide MSC mechanosensing in 3D hydrogels *via* Hippo signaling activation, which is mediated by transcriptional co-activators Yes-associated protein (YAP)/transcriptional coactivator with a PDZ-binding domain (TAZ) and regulates fate decision of MSCs 23. However, whether and how 3D culture may preserve the immunomodulation of MSCs against continuous passages remain elusive.

In this study, we aimed to investigate the potential effects and underlying mechanisms of 3D culture on immunomodulation of MSCs which may benefit their therapeutic performance despite extensive expansion. We selected IBD and T1D to investigate MSC immunotherapy, which are among the most prevalent autoimmune diseases and are widely adopted for this research purpose 24, 25, and well-established mouse models of dextran sulfate sodium (DSS)-induced colitis and streptozotocin (STZ)-induced T1D were used. It has been reported that IBD and T1D share common pathological characteristics in immune hyperactivation and prolonged inflammation, and that genetic loci overlapped between IBD and T1D have putative effects on the development of these diseases, leading to bidirectional positive links between IBD and T1D 26-28. MSCs were examined at the fifth (P5) and the fifteenth (P15) passages, and 3D culture was conducted by Matrigel incorporation. We reveal that MSCs lose therapeutic immunomodulatory effects after extensive expansion to P15, which is counteracted by 3D preconditioning thus safeguarding the MSC function to modulate T-cell subsets, suppress infiltration of pro-inflammatory T cells in colon and pancreas tissues, ameliorate inflammation, and alleviate murine colitis and T1D. We further show that 3D culture triggers transcriptomic reprogramming of MSCs toward a YAP1-marked state, Hippo signaling pathway-upregulated state with promoted release of the anti-inflammatory cytokine, transforming growth factor-beta1 (TGF-β1), and that chemical regulation of YAP1 by clinically relevant drugs, verteporfin (VP) and prostaglandin E2 (PGE2), affects TGF-β1 expression and the immunomodulatory capacity of MSCs during dimensional culture. Taken together, these findings first unravel YAP1-based dimensional and chemical coordination of immunoregulation of expanded MSCs, shedding light on precisely controlled translational application.

## Results

### MSCs fail to treat DSS-induced colitis and STZ-induced T1D after extensive passages

**Figure 1:**
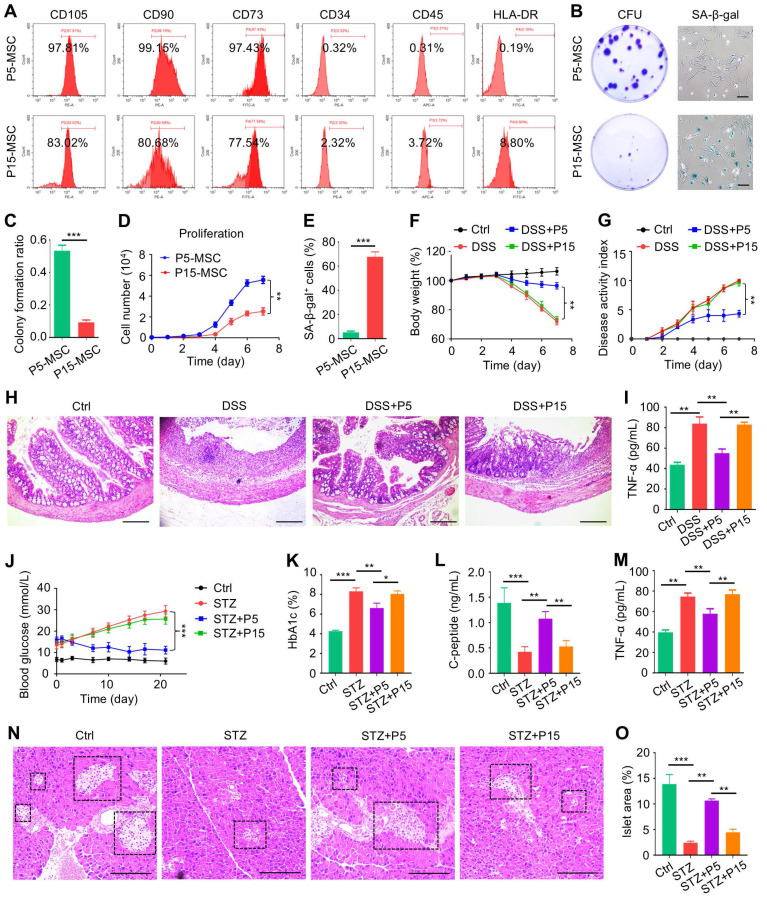
** MSCs fail to treat DSS-induced colitis and STZ-induced T1D after extensive passages.** (A) Flow cytometric analysis of surface markers of MSCs. (B) Colony formation of MSCs analyzed by crystal violet staining, and cell senescence of MSCs analyzed by SA-β-gal staining. Scale bars = 100 μm. (C) Quantification of colony forming units of MSCs over total seeded cells. (D) Proliferation rate of MSCs analyzed by CCK8. (E) Quantification of senescent percentages of MSCs. (F) Body weight of mice recorded during the experimental period. DSS was administered at 2.5% (m/v) in drinking water from Day 1 to Day 5. One million MSCs were infused *via* caudal vein at Day 3. (G) Disease activity index of mice recorded during the experimental period. (H) H&E staining showing the tissue histopathology of colon. Scale bars = 250 μm. (I) ELISA analysis of serum TNF-α levels at Day 7. (J) Random blood glucose levels recorded during the experimental period. Intraperitoneal injections of 50 mg/kg STZ were performed daily from Day 1 to Day 5. One million MSCs were infused *via* caudal vein at Day 7 and Day 14. (K) Serum HbA1c levels detected at Day 21. (L) ELISA analysis of serum C-peptide levels at Day 7. (M) ELISA analysis of serum TNF-α levels at Day 21. (N) H&E staining showing the tissue histopathology of pancreas. Black dashed brackets indicating pancreas islets. Scale bars = 250 μm. (O) Quantification of pancreas islet area percentages. *N* = 3 per group. Mean ± SD. *, *P* < 0.05; **, *P* < 0.01; ***, *P* < 0.001. Two-tailed unpaired Student's *t* test (C-E) or one-way ANOVA with Bonferroni's post-hoc test (F-O).

To begin, we respectively characterized P5-MSCs and P15-MSCs according to their surface proteins. Expectedly, MSCs at both passages highly expressed typical positive markers, such as CD105, CD90 and CD73, whereas they were rarely exposed with hematopoietic antigens, including CD34, CD45 and human leukocyte antigen DR (HLA-DR) (Figure 1A). Moreover, P15-MSCs were confirmed to grow diminished colony formation units (CFU), show apparent senescence-associated beta-galactosidase (SA-β-gal) activity, and demonstrate suppressed proliferation compared to P5-MSCs, indicating impaired stemness and replicative senescence (Figure 1B-E). Importantly, upon intravenous infusion, P15-MSCs failed to protect body weight loss or alleviate colitis disease symptoms (diarrhea and hematochezia) of DSS-treated mice, while P5-MSCs could ameliorate the disease activity (Figure 1F-G). Pathological changes of the colon mucosa confirmed the immunosuppressive effect of P5-MSCs, rather than P15-MSCs, in treating colitis, which was also supported by enzyme-linked immunosorbent assay (ELISA) analysis of the serum pro-inflammatory cytokine, tumor necrosis factor-alpha (TNF-α) (Figure 1H-I). To further verify the immunomodulatory function, P5-MSCs and P15-MSCs were systemically administrated to treat STZ-challenged T1D mice. Examination of the random blood glucose levels revealed that only P5-MSCs were effective in controlling hyperglycemia of T1D mice (Figure 1J). Serum analyses demonstrated that P5-MSCs, but not P15-MSCs, reduced the glycated hemoglobin (HbA1c) level, with promoting the C-peptide concentration (reflecting islet β-cell function) and suppressing the TNF-α level of STZ-induced T1D mice (Figure 1K-M). Histological evaluation of the pancreas islets was additionally performed, which demonstrated a remarkable rescue of islet area by P5-MSCs, with only marginal effect exerted by P15-MSCs (Figure 1N-O). Taken together, these results suggested that MSCs fail to treat DSS-induced colitis and STZ-induced T1D after extensive passages.

### 3D culture promotes diminished immunoregulatory capability of MSCs after extensive passages

**Figure 2:**
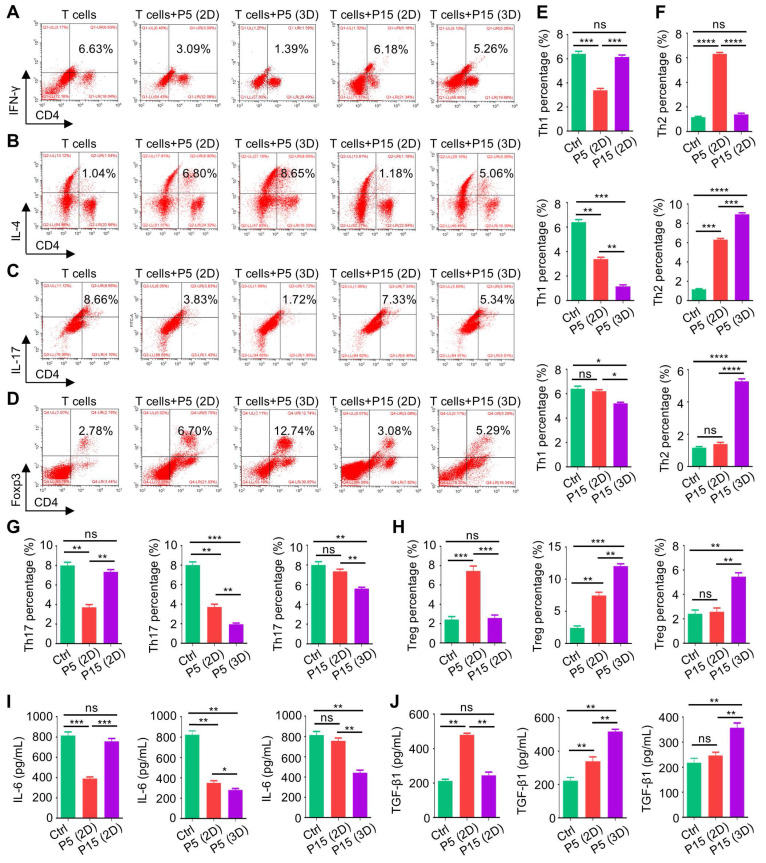
** Three-dimensional culture promotes diminished immunoregulatory capability of MSCs after extensive passages.** (A) Flow cytometric analysis showing CD4^+^IFN-γ^+^ Th1 cell percentages after T cells co-culture with MSCs for 3 days at the proportion of 10:1. MSCs were preconditioned on TCP (2D) or Matrigel (3D) across passages. (B) Flow cytometric analysis showing CD4^+^IL-4^+^ Th2 cell percentages after T cells co-culture with MSCs. (C) Flow cytometric analysis showing CD4^+^IL-17^+^ Th17 cell percentages after T cells co-culture with MSCs. (D) Flow cytometric analysis showing CD4^+^Foxp3^+^ Treg cell percentages after T cells co-culture with MSCs. (E) Quantification of Th1 cell percentages. (F) Quantification of Th2 cell percentages. (G) Quantification of Th17 cell percentages. (H) Quantification of Treg cell percentages. (I) ELISA analysis of IL-6 levels in the conditioned media. (J) ELISA analysis of TGF-β1 levels in the conditioned media. *N* = 3 per group. Mean ± SD. *, *P* < 0.05; **, *P* < 0.01; ***, *P* < 0.001; ****, *P* < 0.0001; ns, *P* > 0.05. One-way ANOVA with Bonferroni's post-hoc test.

Next, we investigated that whether 3D culture would benefit the immunomodulatory function of MSCs. We adopted a preconditioning experimental system where MSCs started at P3 were seeded in Matrigel before harvest at P5 or P15 for following tests, while the 2D-cultured MSCs as the control group were maintained on the tissue culture plastic (TCP) surface during the study. 3D-preconditioned MSCs were then tested for functionality in the routine 2D condition to evaluate whether the acquired properties would be maintained despite separation from the 3D environment. Not surprisingly, 3D culture did not alter the surface marker profiles of MSCs (Figure S1A). However, MSCs preconditioned in the 3D environment indeed showed enhanced clonogenicity, alleviated cell senescence and promoted proliferation when tested at both P5 and P15 (Figure S1B-F). Then, to evaluate the immunomodulatory function of MSCs, we used purified CD3^+^ T cells for a direct co-culture with MSCs and examined different sub-populations of T cells, as well as cytokines in the conditioned media. Flow cytometric outcomes showed that continuous culture in the 2D environment led to dramatic decline of the MSC capability to suppress CD4^+^interferon gamma (IFN-γ)^+^ T helper 1 (Th1) and CD4^+^interleukin 17 (IL-17)^+^ Th17 cell percentages after extensive (P15, compared to P5) passages, which also lost the capacity to promote CD4^+^IL-4^+^ Th2 and CD4^+^Forkhead box protein P3 (Foxp3)^+^ T regulatory (Treg) cell differentiation (Figure 2A-D). Interestingly, 3D preconditioning not only enhanced immunomodulation of MSCs at P5 (after three passages of 3D culture) but also rescued the diminished MSC functionality at P15 (after thirteen passages of 3D culture) (Figure 2A-D). Statistical analyses confirmed these changes, showing beneficial effects of 3D culture on immunoregulatory performance of both P5-MSCs and P15-MSCs (Figure 2E-H). Furthermore, ELISA detection of the pro-inflammatory cytokine, IL-6, and the anti-inflammatory cytokine, TGF-β1, in the co-cultured media supernatant, verified that P15-MSCs showed robust anti-inflammatory effects after 3D preconditioning (Figure 2I-J). Taken together, these findings suggested that 3D culture promotes diminished immunoregulatory capability of MSCs after extensive passages.

### 3D preconditioning benefits therapeutic effects of extensively passaged MSCs on colitis and T1D

**Figure 3:**
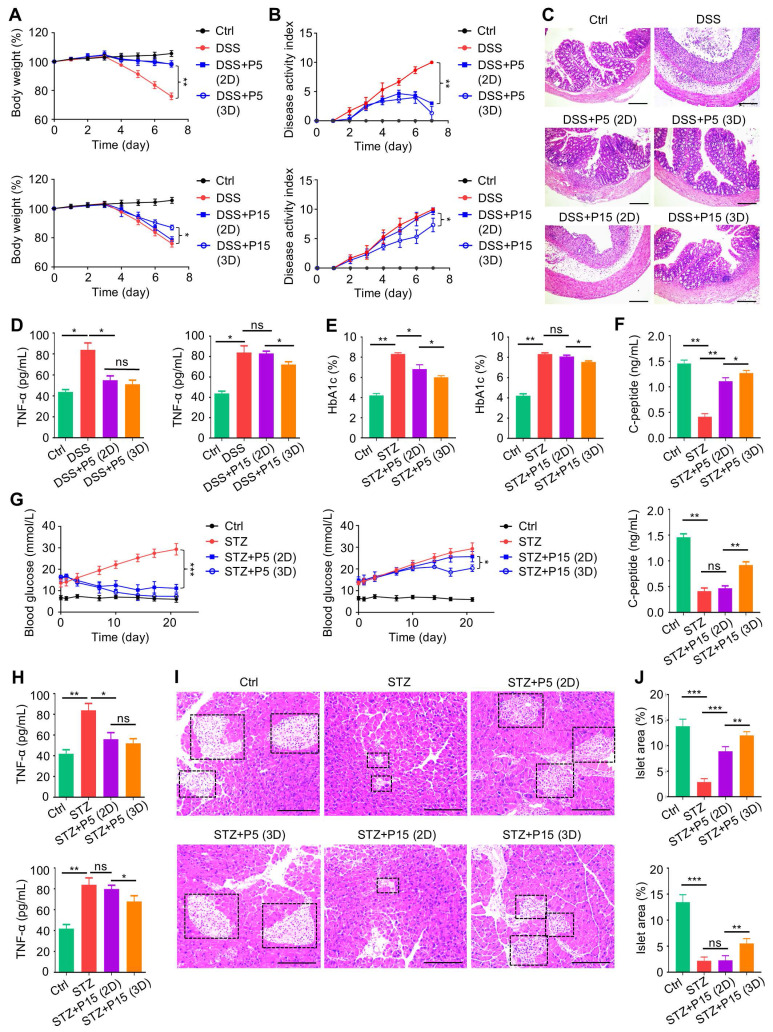
** Three-dimensional preconditioning benefits therapeutic effects of extensively passaged MSCs on colitis and T1D.** (A) Body weight of mice. DSS was administered at 2.5% (m/v) in drinking water from Day 1 to Day 5. One million MSCs were infused *via* caudal vein at Day 3. MSCs were preconditioned on TCP (2D) or Matrigel (3D) across passages. (B) Disease activity index of mice. (C) H&E staining showing the tissue histopathology of colon. Scale bars = 250 μm. (D) ELISA analysis of serum TNF-α levels at Day 7. (E) Serum HbA1c levels detected at Day 21. Intraperitoneal injections of 50 mg/kg STZ were performed daily from Day 1 to Day 5. One million MSCs were infused *via* caudal vein at Day 7 and Day 14. MSCs were preconditioned on TCP (2D) or Matrigel (3D) across passages. (F) ELISA analysis of serum C-peptide levels at Day 7. (G) Random blood glucose levels recorded during the experimental period. (H) ELISA analysis of serum TNF-α levels at Day 21. (I) H&E staining showing the tissue histopathology of pancreas. Black dashed brackets indicating pancreas islets. Scale bars = 250 μm. (J) Quantification of pancreas islet area percentages. *N* = 3 per group. Mean ± SD. *, *P* < 0.05; **, *P* < 0.01; ***, *P* < 0.001; ns, *P* > 0.05. One-way ANOVA with Bonferroni's post-hoc test.

We then evaluated that whether 3D preconditioning would improve the therapeutic efficacy of extensively passaged MSCs in treating autoimmune diseases. We discovered that while 3D preconditioning did not significantly promote ameliorative effects of P5-MSCs on colitis, it did safeguard the immunomodulatory effects of P15-MSCs despite passages, showing protected body weight and alleviated disease activity after infusion of 3D-preconditioned P15-MSCs into DSS-treated mice (Figure 3A-B). These findings were supported by the colon histological examinations, exhibiting improved mucosal structure after infusion of 3D-preconditioned P15-MSCs into DSS-treated mice (Figure 3C). Also, serum analysis of TNF-α levels confirmed the preserved *in vivo* immunoregulatory capability of P15-MSCs after 3D culture (Figure 3D). With regard to the T1D treatment, serum analyses demonstrated that 3D preconditioning promoted both capacities of P5-MSCs and P15-MSCs to reduce HbA1c levels and elevate C-peptide concentrations (Figure 3E-F), while the significant improvement on random blood glucose levels was only seen after T1D mice injected with 3D-preconditioned P15-MSCs (Figure 3G). Moreover, serum analysis of TNF-α levels verified the therapeutic immunoregulatory capability of P15-MSCs after 3D culture (Figure 3H). While histological analysis of pancreas islets still demonstrated certain beneficial effects of 3D culture on P5-MSCs to promote islet recovery, the improvement of P15-MSCs by 3D preconditioning to retard the islet alterations was definite (Figure 3I-J). Taken together, these findings indicated that 3D preconditioning benefits therapeutic effects of extensively passaged MSCs on colitis and T1D.

### 3D preconditioning empowers extensively passaged MSCs to suppress infiltration of pro-inflammatory T cells in colon and pancreas tissues

**Figure 4:**
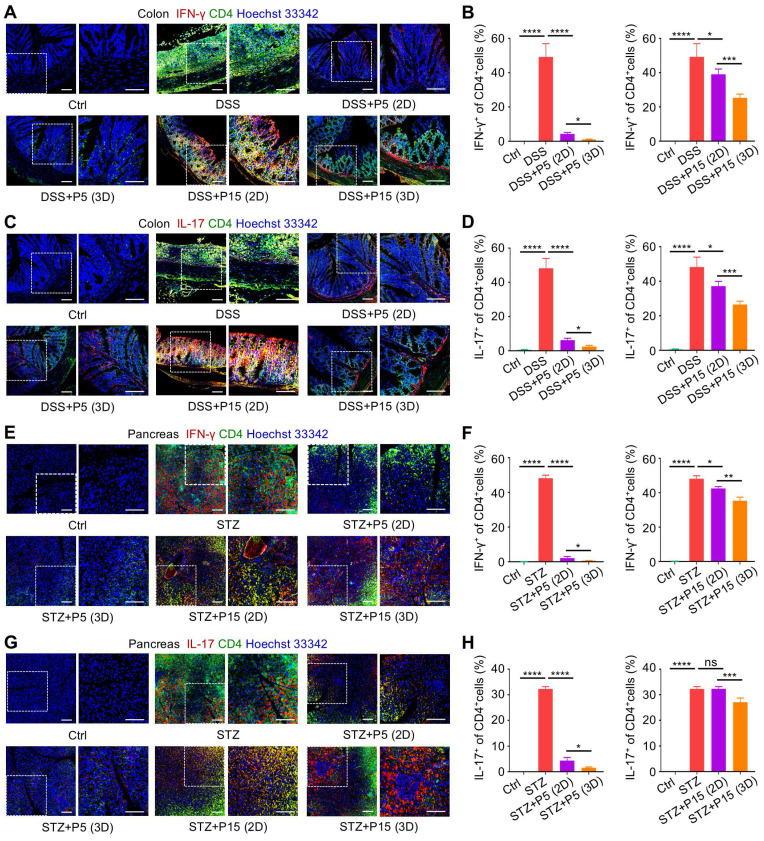
** Three-dimensional preconditioning promotes effects of extensively passaged MSCs to suppress infiltration of pro-inflammatory T-cell subsets in colon and pancreas tissues.** (A) IF staining of IFN-γ (red) with CD4 (green) in colon tissues counterstained by Hoechst 33342 (blue). MSCs were preconditioned on TCP (2D) or Matrigel (3D) across passages and were used to treat DSS-challenged mice. Scale bars = 100 μm. (B) Quantification of IFN-γ-positive percentages in total CD4-positive cells in colon. (C) IF staining of IL-17 (red) with CD4 (green) in colon tissues counterstained by Hoechst 33342 (blue). Scale bars = 100 μm. (D) Quantification of IL-17-positive percentages in total CD4-positive cells in colon. (E) IF staining of IFN-γ (red) with CD4 (green) in pancreas tissues counterstained by Hoechst 33342 (blue). 2D- and 3D-preconditioned MSCs across passages were used to treat STZ-challenged mice. Scale bars = 100 μm. (F) Quantification of IFN-γ-positive percentages in total CD4-positive cells in pancreas. (G) IF staining of IL-17 (red) with CD4 (green) in pancreas tissues counterstained by Hoechst 33342 (blue). Scale bars = 100 μm. (H) Quantification of IL-17-positive percentages in total CD4-positive cells in pancreas. *N* = 3 per group. Mean ± SD. *, *P* < 0.05; **, *P* < 0.01; ***, *P* < 0.001; ****, *P* < 0.0001; ns, *P* > 0.05. One-way ANOVA with Bonferroni's post-hoc test.

Next, we investigated whether recipient T cells were indeed regulated by infused MSCs *in vivo*. After harvest of the colon tissues, immunofluorescence (IF) staining was performed to evaluate T-cell infiltration into target tissues in the DSS-induced colitis model. We discovered that in colitis, pro-inflammatory Th1 and Th17 T-cell subsets, marked respectively by double positive CD4^+^IFN-γ^+^ and CD4^+^IL-17^+^ staining, accumulated in colon, which were dramatically inhibited by infused P5-MSCs cultured in both 2D and 3D environments (Figure 4A-D). Interestingly, P15-MSCs from 2D culture were less effective in control of pro-inflammatory T-cell infiltration, although slight efficacy was detected, which was not enough to prevent disease progression (Figure 4A-D). 3D-preconditioned P15-MSCs, nevertheless, were therapeutically competent in suppressing pro-inflammatory T-cell infiltration in colon, which reduced Th1 and Th17 cells more effectively compared to 2D-cultured P15-MSCs (Figure 4A-D). Regarding T cells in T1D, still pro-inflammatory Th1 and Th17 T-cell subsets were infiltrated in pancreas tissues, which were significantly inhibited by therapeutically infused P5-MSCs cultured in both 2D and 3D environments (Figure 4E-H). Importantly, 3D preconditioning enhanced capacity of P15-MSCs to suppress pro-inflammatory T-cell infiltration in pancreas, which was corresponded to the therapeutic efficacy observed, while 2D-cultured P15-MSCs revealed insufficient capability of *in vivo* immunoregulation (Figure 4E-H). Taken together, these findings suggested that 3D preconditioning empowers expanded MSCs to suppress infiltration of pro-inflammatory T cells in target tissues.

### 3D culture induces transcriptomic reprogramming of MSCs toward a YAP1-marked state with TGF-β1 upregulation

**Figure 5:**
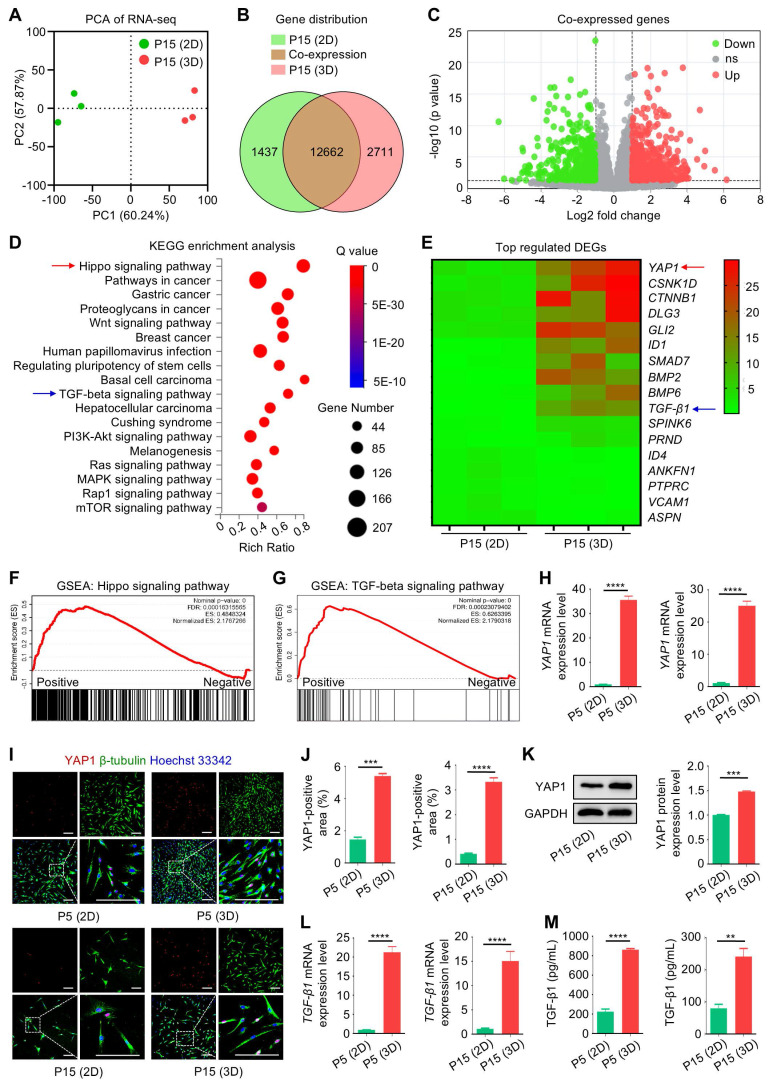
** Three-dimensional culture induces transcriptomic reprogramming of MSCs toward a YAP1-marked state with TGF-β1 upregulation.** (A) Graph showing PCA of RNA-seq data. MSCs were cultured on TCP (2D) or Matrigel (3D) across passages. (B) Venn diagram depicting gene distribution detected in RNA-seq. (C) Volcano plot showing DEGs of 3D over 2D MSCs after extensive passages. (D) KEGG enrichment analysis of DEGs of 3D over 2D MSCs after extensive passages. Red and blue arrows respectively indicate Hippo and TGF-β signaling pathways. (E) Heatmap showing top regulated DEGs of 3D over 2D MSCs after extensive passages. Red and blue arrows respectively indicate *YAP1* and *TGF-β1*. (F) GSEA assay of DEGs regarding the Hippo signaling pathway. (G) GSEA assay of DEGs regarding the TGF-β signaling pathway. (H) qRT-PCR analysis of *YAP1* mRNA expression in MSCs. (I) IF staining of YAP1 (red) with β-tubulin (green) in MSCs counterstained by Hoechst 33342 (blue). MSCs were preconditioned on TCP (2D) or Matrigel (3D) across passages. Scale bars = 200 μm. (J) Quantification of YAP1-positive area percentages. (K) WB analysis of YAP1 protein expression in MSCs with quantification. (L) qRT-PCR analysis of *TGF-β1* mRNA expression in MSCs. (M) ELISA analysis of TGF-β1 levels in the conditioned media of MSCs. *N* = 3 per group. Mean ± SD. **, *P* < 0.01; ***, *P* < 0.001; ****, *P* < 0.0001. Two-tailed unpaired Student's *t* test.

Next, we investigated how 3D culture may affect the functionality of MSCs during passages. To this end, we performed RNA sequencing (RNA-seq) analysis of the transcriptomic profiles of 2D- and 3D-cultured MSCs at P15. Principal component analysis (PCA) of the RNA-seq data demonstrated that 2D- and 3D-cultured P15-MSCs were differentially distributed in the gene expression signature (Figure 5A), which showed 12,662 overlapping genes in the Venn diagram (Figure 5B). Of the co-expressed genes, 1,138 were upregulated in 3D-cultured P15-MSCs compared to the 2D counterparts, while 1,088 were down-regulated among differentially expressed genes (DEGs) (Figure 5C). For the potential function of DEGs, enrichment analysis of DEGs by the Kyoto Encyclopedia of Genes and Genomes (KEGG) database suggested multiple related terms, the most prominent of which was the “Hippo signaling pathway”, also including the “Wnt signaling pathway”, the “Regulating pluripotency of stem cells”, and the immunomodulation-relevant term “TGF-beta signaling pathway”, among others (Figure 5D). Furthermore, by checking the top regulated DEGs in the transcriptomic data, we found *YAP1*, the vital downstream effector of the Hippo signaling pathway, demonstrated the highest level of upregulation in 3D-cultured over 2D-cultured P15-MSCs (Figure 5E). Besides, TGF-β1 was among the top 10 upregulated DEGs in 3D-cultured over 2D-cultured P15-MSCs (Figure 5E). Gene set enrichment analysis (GSEA) confirmed upregulation of both Hippo and TGF-beta signaling pathways in 3D-cultured over 2D-cultured P15-MSCs (Figure 5F-G). Moreover, quantitative real-time polymerase chain reaction (qRT-PCR) analysis verified the changes of *YAP1* mRNA expression in MSCs, which exhibited beyond 20 folds of increase during 3D culture (Figure 5H). We have also performed IF staining to examine the YAP1 expression in MSCs, together with the cellular phenotypes identified by IF co-staining of β-tubulin, the key component of the microtubule cytoskeleton. Results demonstrated that while P15-MSCs were less positive of YAP1 and less spindle in shape than P5-MSCs, 3D preconditioning significantly promoted the YAP1 positive rate of both P5-MSCs and P15-MSCs with stretching cell shapes (Figure 5I-J). The YAP1 upregulation in 3D-cultured MSCs was additionally verified by the Western blot (WB) assay (Figure 5K). Notably, enhanced* TGF-β1* transcription with promoted release of TGF-β1 by 3D-cultured MSCs were revealed (Figure 5L-M). Taken together, our findings indicated that 3D culture induces transcriptomic reprogramming of MSCs toward a YAP1-marked state with TGF-β1 upregulation.

### Chemical regulation of YAP1 affects TGF-β1 expression and the immunomodulatory capacity of MSCs

**Figure 6:**
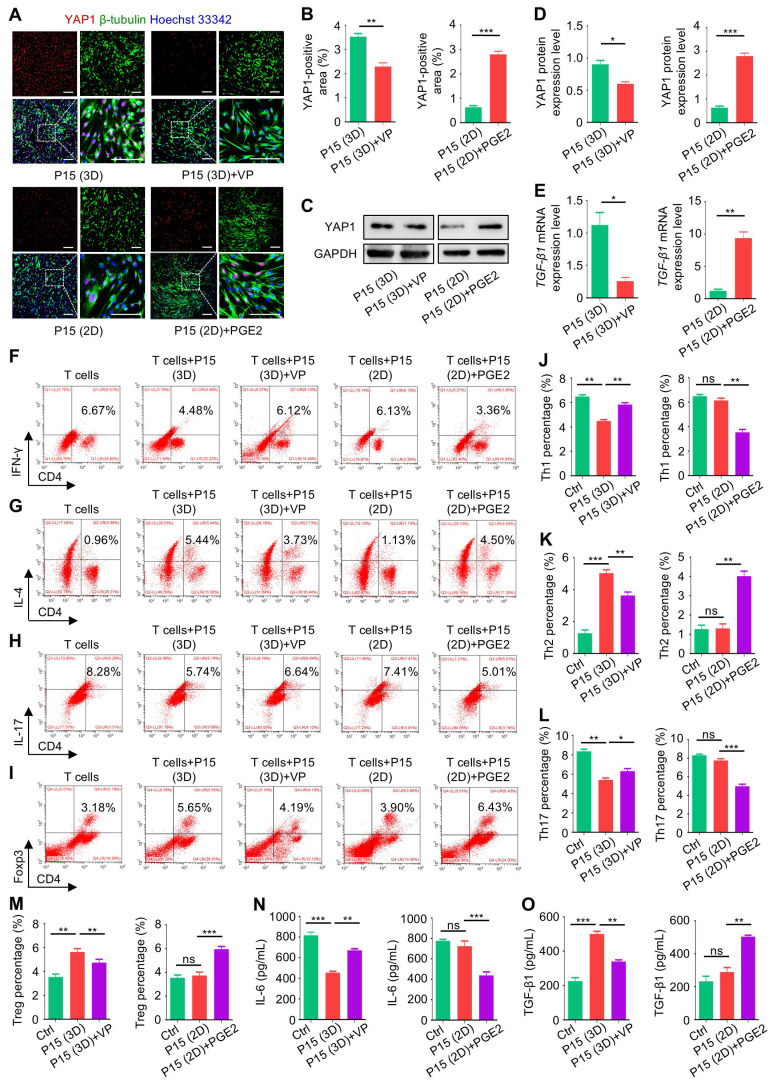
** Chemical regulation of YAP1 affects TGF-β1 expression and the immunomodulatory capacity of MSCs.** (A) IF staining of YAP1 (red) with β-tubulin (green) in MSCs counterstained by Hoechst 33342 (blue). MSCs were preconditioned on TCP (2D) or Matrigel (3D) across passages, then treated with or without 10 µM VP and PGE2 for 48 h before testing. Scale bars = 200 μm. (B) Quantification of YAP1-positive area percentages. (C) WB analysis of YAP1 protein expression in MSCs. (D) Quantification of WB analysis of YAP1 protein expression. (E) qRT-PCR analysis of *TGF-β1* mRNA expression in MSCs. (F) Flow cytometric analysis showing CD4^+^IFN-γ^+^ Th1 cell percentages after T cells co-culture with MSCs for 3 days at the proportion of 10:1. (G) Flow cytometric analysis showing CD4^+^IL-4^+^ Th2 cell percentages after T cells co-culture with MSCs. (H) Flow cytometric analysis showing CD4^+^IL-17^+^ Th17 cell percentages after T cells co-culture with MSCs. (I) Flow cytometric analysis showing CD4^+^Foxp3^+^ Treg cell percentages after T cells co-culture with MSCs. (J) Quantification of Th1 cell percentages. (K) Quantification of Th2 cell percentages. (L) Quantification of Th17 cell percentages. (M) Quantification of Treg cell percentages. (N) ELISA analysis of IL-6 levels in the conditioned media. (O) ELISA analysis of TGF-β1 levels in the conditioned media. *N* = 3 per group. Mean ± SD. *, *P* < 0.05; **, *P* < 0.01; ***, *P* < 0.001; ns, *P* > 0.05. Two-tailed unpaired Student's *t* test (B-E) or one-way ANOVA with Bonferroni's post-hoc test (J-O).

Next, we investigated that whether YAP1 was indeed the key mediator of 3D cultural effects on MSCs and that whether chemical modulation of YAP1 would affect the immunomodulatory function of MSCs. We selected VP, a specific inhibitor of YAP1 29, and PGE2, which was reported to increase the transcriptional activity of YAP1 30. With a translational perspective, VP and PGE2 are both clinically approved drugs 31, 32. IF staining and WB analyses together confirmed that application of VP in 3D culture suppressed YAP1 expression in P15-MSCs, whereas PGE2 treatment promoted YAP1 expression in P15-MSCs despite 2D culture (Figure 6A-D). Furthermore, TGF-β1 transcription was also affected by chemical regulation of YAP1, indicating its downstream of Hippo signaling (Figure 6E).

For the immunoregulatory capability, flow cytometric analyses revealed that chemical inhibition of YAP1 by VP led to abolished effects of 3D preconditioning on P15-MSCs, resulting in diminished efficacies to suppress Th1 and Th17 cell populations or promote Th2 and Treg cell differentiation from purified CD3^+^ T cells (Figure 6F-I). On the other hand, chemical stimulation of YAP1 by PGE2 enhanced immunoregulatory function of P15-MSCs despite 2D culture (Figure 6F-I). Statistical analyses verified these immunophenotypic changes (Figure 6J-M), which were further supported by ELISA analyses of IL-6 and TGF-β1 in the co-cultured conditioned media of MSCs and T cells (Figure 6N-O). Collectively, these findings suggested that chemical regulation of YAP1 affects immunomodulatory capacity of MSCs.

### MSC therapy against colitis and T1D is dependent on chemical regulation of YAP1 in dimensional culture

**Figure 7:**
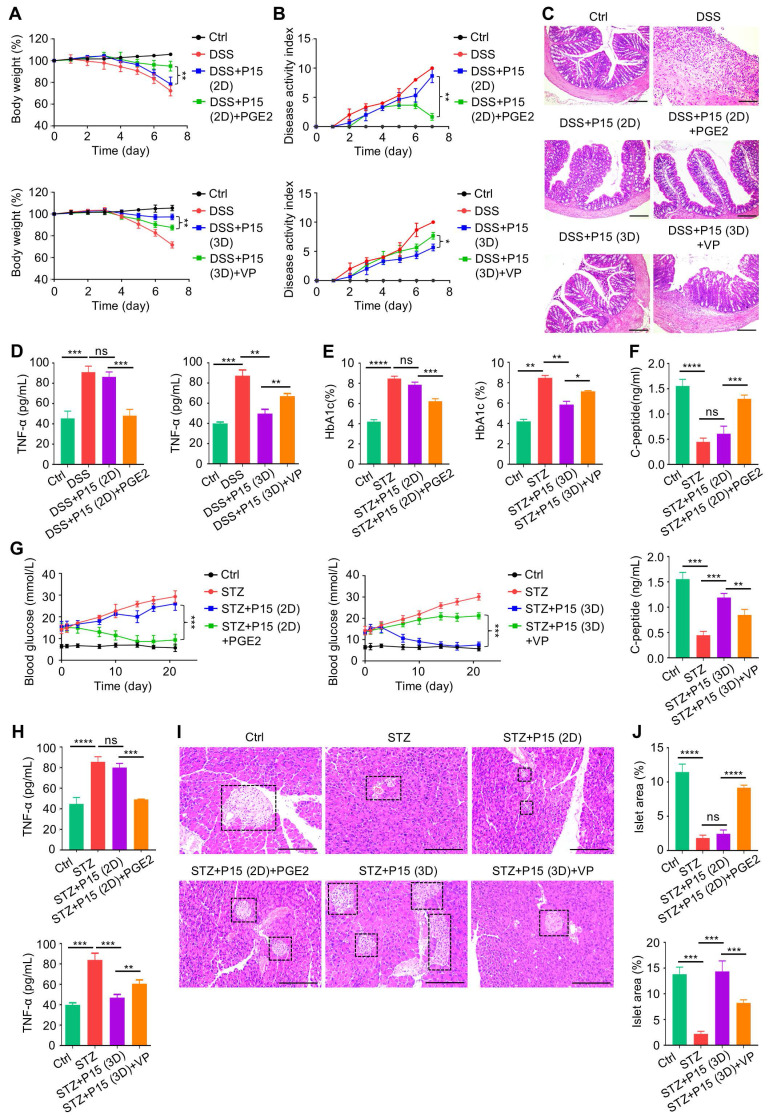
** MSC therapy against colitis and T1D is dependent on chemical regulation of YAP1 in dimensional culture.** (A) Body weight of mice. DSS was administered at 2.5% (m/v) in drinking water from Day 1 to Day 5. One million MSCs were infused *via* caudal vein at Day 3. MSCs were preconditioned on TCP (2D) or Matrigel (3D) across passages, then treated with or without 10 µM VP and PGE2 for 48 h before testing. (B) Disease activity index of mice. (C) H&E staining showing the tissue histopathology of colon. Scale bars = 250 μm. (D) ELISA analysis of serum TNF-α levels at Day 7. (E) Serum HbA1c levels detected at Day 21. Intraperitoneal injections of 50 mg/kg STZ were performed daily from Day 1 to Day 5. One million MSCs were infused *via* caudal vein at Day 7 and Day 14. MSCs were preconditioned on Matrigel (3D) across passages, then treated with or without 10 µM VP and PGE2 for 48 h before testing. (F) ELISA analysis of serum C-peptide levels at Day 7. (G) Random blood glucose levels recorded during the experimental period. (H) ELISA analysis of serum TNF-α levels at Day 21. (I) H&E staining showing the tissue histopathology of pancreas. Black dashed brackets indicating pancreas islets. Scale bars = 250 μm. (J) Quantification of pancreas islet area percentages. *N* = 3 per group. Mean ± SD. *, *P* < 0.05; **, *P* < 0.01; ***, *P* < 0.001; ****, *P* < 0.0001; ns, *P* > 0.05. One-way ANOVA with Bonferroni's post-hoc test.

Finally, we investigated that whether chemical regulation of YAP1 affects therapeutic efficacy of MSCs and underlies the effects of dimensional culture. We discovered that PGE2 treatment during 2D culture promoted therapeutic performance of P15-MSCs on DSS-induced colitis, as shown by better preserved body weight and ameliorated disease activity (Figure 7A-B). On the other hand, VP-treated P15-MSCs reduced the therapeutic benefits on colitis obtained during 3D-preconditioning (Figure 7A-B). Colon histology by hematoxylin and eosin (H&E) staining confirmed the gross observations, which was further supported by ELISA analysis of TNF-α levels, revealing affected immunomodulation* in vivo* by chemical regulation of YAP1 (Figure 7C-D). For the STZ-induced T1D model, peripheral Hb1Ac together with C-peptide concentrations demonstrated that PGE2 treatment during 2D culture promoted therapeutic effects of P15-MSCs, whereas 3D-preconditioned P15-MSCs were decreased in therapeutic performance to control hyperglycemia and promote islet function after VP inhibition of YAP1 (Figure 7E-F). Moreover, PGE2-treated 2D-cultured P15-MSCs were competent in T1D therapy, while VP treatment led to lost capacity of 3D-preconditioned P15-MSCs to suppress random blood glucose levels of T1D mice (Figure 7G). ELISA analysis of TNF-α levels and histological examinations of the pancreas islets verified the findings, exhibiting that 2D-cultured P15-MSCs were boosted by PGE2 in therapeutic immunomodulation and that VP-treated P15-MSCs were not well qualified to reduce inflammation or restore the tissue health despite MSCs preconditioned in the 3D environment (Figure 7H-J). Together, these data suggested that MSC therapy against colitis and T1D is dependent on chemical regulation of YAP1 in dimensional culture.

**Figure 8:**
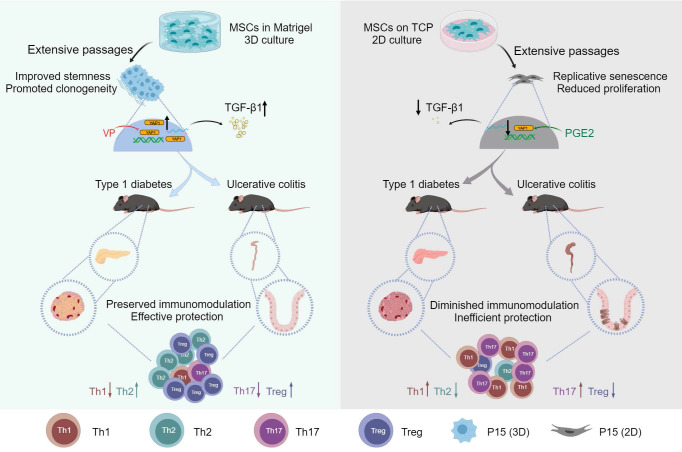
** Graphical conclusion of this study.** MSCs lose therapeutic immunomodulatory effects against DSS-induced colitis and STZ-induced T1D after extensive passages, which is counteracted by 3D preconditioning. Transcriptomic reprogramming mediates effects of 3D culture on MSC immunomodulation through YAP1 regulation of TGF-β1 expression, and YAP1 contributes to dimensional and chemical coordination of expanded MSC immunoregulation. Created with MedPeer (medpeer.cn).

In brief summary, MSCs lose therapeutic immunomodulatory effects against DSS-induced colitis and STZ-induced T1D after extensive passages toward replicative senescence, which is counteracted by 3D preconditioning. Transcriptomic reprogramming mediates effects of 3D culture on MSC immunomodulation through YAP1 regulation of TGF-β1 expression, and YAP1 contributes to dimensional and chemical coordination of expanded MSC immunoregulation (Figure 8). These findings shed light on precisely controlled translational application of MSC immunotherapy.

## Discussion

MSCs have potent immunomodulatory capability and have shown promise in therapeutic use 3, 4. However, clinical application of MSCs is still hindered by compromised cell functionality and insufficient therapeutic efficacy at some cases 6-9, 33-40. Here, using well-established mouse models of DSS-induced colitis and STZ-induced T1D, we demonstrate that MSCs lose therapeutic immunomodulatory effects after expansion to P15. Intriguingly, 3D preconditioning of MSCs promotes diminished immunoregulatory capability despite passages. Mechanistically, 3D culture provokes transcriptomic reprogramming of MSCs toward a YAP1-marked, Hippo signaling pathway-upregulated state with promoted release of TGF-β1. Importantly, chemical regulation of YAP1 by VP and PGE2 affects TGF-β1 expression and the immunomodulatory capacity of MSCs during dimensional culture. Together, these findings unravel YAP1-based dimensional and chemical coordination of expanded MSC immunoregulation, shedding light on precisely controlled translational application.

MSCs are a kind of tissue stem cells originally identified from the bone marrow stroma based on their self-renewal and colony formation capabilities, which are later on revealed to have multi-differentiation and immunomodulation potentials to create favorable environments and enhance tissue repair 1, 4, 41. Interestingly, the immunoregulation of MSCs is not intrinsic, but inducible by inflammation and cell-cell interaction, which is also dynamically influenced by the various factors involved in a complex cell-host interplay 42-44. We have previously shown that MSC immunomodulation in treating T1D-related osteoporosis is governed by a glycemic microenvironment, unraveling the recipient component regulating therapeutic effects of MSCs 45. We have also documented that long-term *in vitro* expansion diminishes MSC stemness and causes failure in regenerative and immunosuppressive therapy, confirming the donor cell-dependent regulatory effects 16. It is widely acknowledged that continuous passages result in replicative senescence of MSCs, which can be attributed to telomere attrition, mitochondrial compromise and transcriptomic alterations 14, 46, 47. In this study, we further demonstrate that extensive expansion of MSCs to P15 reduces the immunomodulatory capability, which might be due to a shifted secretory profile away from anti-inflammation *via* decreased TGF-β1 release. Indeed, TGF-β1 is an established cytokine regulating T-cell differentiation, which can also be secreted by MSCs for immunosuppression 48, 49. High-throughput screening of other potential cytokines involved in altered MSC-T cell communication after cultural expansion of MSCs are expected to be investigated in future studies.

To maintain functionality of MSCs in non-physiological condition *in vitro*, it is essential to mimic the *in vivo* microenvironment through 3D construction 22. The encapsulation of MSCs, by entrapping the viable cells in a 3D semi-permeable hydrogel matrix, is one of the simple approach to introduce a 3D microenvironment 19, 50. It has been established that cells show different morphological and functional phenotype in 2D and 3D microenvironments, and that MSCs demonstrate enhanced proliferation, differentiation and angiogenic regulation during 3D culture 19. It has also been reported that 3D culture strengthens secretion of biological factors and benefits the immunomodulation of MSCs 20, 51-53. In this study, on the basis of these previous studies, we further show that 3D preconditioning of MSCs in Matrigel promotes the therapeutic immunomodulatory capability, which counteracts the diminished effects introduced by extensive passages. Notably, P5-MSCs are competent in therapeutic immunomodulation, suggesting the 2D plain culture largely sufficient in this context, while they still show enhanced immunoregulatory capacity after 3D preconditioning. YAP1-mediated 3D-reconditioning effects are more prominent on P15-MSCs, which are reasonable, because that extensive passages dampen the donor cell functionality, making them increasingly in demand for dimensional and chemical stimulation. The findings are important as they provide a safe and effective method to preserve therapeutic potential of MSCs while retaining expansion, and suggest that the 3D effects might be “memorized” once established despite away from the 3D microenvironment during translational application. Whether epigenetic mechanisms potentially contribute to this process will be interesting to examine.

IBD is a chronic relapsing disease that includes Crohn's disease and ulcerative colitis, the global incidence and prevalence of which, particularly in Asian countries, have been increasing 54, 55. With the pathogenesis of IBD remaining unclear, up to 40% of patients with IBD failed to show ideal therapeutic results to current immunosuppressive agents 56, 57. T1D is an autoimmune disease characterized by the destruction of insulin-producing pancreas β cells, leading to progressive insulin deficiency and hyperglycemia, which causes complications, such as cardiovascular diseases, neuropathy, nephropathy and retinopathy 58-60. Current insulin application or pancreas islet transplantation is limited to achieve the curative goal of T1D due to insufficient effectiveness or donor scarcity 52. Here, we show that intravenous infusion of 3D-preconditioned, extensively passaged MSCs serves as feasible therapeutic for both colitis and T1D, which is regulated by YAP1 expression. The Hippo pathway is initially identified as a suppressor of tissue overgrowth in *Drosophila melanogaster*, and later studies have reported that the general components and functions of the pathway are highly conserved in mammals to regulate cell proliferation, survival and differentiation 61. Interestingly, YAP1 has been documented to have crosstalk with the TGF-β1 signaling, in which YAP1 can potentiate the transactivation of TGF-β1, form protein complexes with the TGF-β signaling effectors Smad2/3, and serve as a transcriptional co-factor of Smad3 62-64. Our findings thus provide basis for optimizing translational application of MSC immunotherapy.

## Methods

### Materials, data and code availability

- The raw sequence data reported in this paper have been deposited in the Genome Sequence Archive 65 in National Genomics Data Center 66, China National Center for Bioinformation / Beijing Institute of Genomics, Chinese Academy of Sciences (GSA-Human: HRA007720) that are publicly accessible at https://ngdc.cncb.ac.cn/gsa-human.
- This paper does not report original code.
- This study did not generate new unique reagents.
- All antibodies, chemicals, commercial assays, medium, oligonucleotides and softwares used were listed (Table S1).
- Any additional information required to reanalyze the data reported in this work is available from the corresponding authors upon reasonable request.

### Mice

Eight-week-old female C57BL/6J mice (weight, 20-22 g) were purchased from the Laboratory Animal Center of the Fourth Military Medical University. Mice were housed in a pathogen-free condition (three mice per cage), maintained on a standard 12-h light-dark cycle, and received normal chow diet and water *ad libitum*. Mice were randomly divided into experimental groups at *N* = 3 per group, and the sample size for the *in vivo* study was set at *N* = 3. Researchers were blinded to the experimental group allocation. All animal experiments were performed in compliance with relevant laws and ethical regulation, following ARRIVE guidelines, and approved by the Animal Care Committee of Xi'an Jiaotong University School of Medicine (permission ID: XJ2006Y039).

### Primary cell cultures

MSCs were isolated from human umbilical cords freshly obtained from women who gave birth in Xi'an No. 4 Hospital. All the donors have signed informed consent to this study, and the experimental procedures of human samples were approved by the Institutional Review Board for Human Subjects Research of Xi'an No. 4 Hospital (reference number: 20190012). Briefly 67, the umbilical cords were carefully removed of blood vessels, chopped into small pieces of 1 mm^3^, and seeded in alpha-minimum essential medium (α-MEM; Gibco, USA, Cat# 12000063) with 10% fetal bovine serum (FBS, Gibco, USA, Cat# 16140071). Five to seven days after seeding, the cells reached 80% confluence, and were then passaged and cultured in α-MEM supplemented with 10% FBS, 100 IU/mL streptomycin (Gibco, USA, Cat# 15140122) and 100 μg/mL penicillin (Gibco, USA, Cat# 15140122). Cells were maintained in a humidified 5% CO_2_ incubator at 37°C. For 2D culture, MSCs were plated directly on TCP and were passaged using 2.5% trypsin (Gibco, USA, Cat# 15050057). For 3D preconditioning, MSCs started at P3 were seeded in Matrigel (Corning, USA, Cat# 356231) and were passaged with the cell recovery solution (Corning, USA, Cat# 354253). MSCs were continuously passaged until P15, and cells at P5 and P15 were used in the following studies.

For isolation of T cells 45, peripheral blood mononucleated cells (PBMNCs) were isolated from fresh whole blood samples collected from healthy donors in Xi'an No. 4 Hospital. All the donors have signed informed consent to this study, and the experimental procedures of human samples were approved by the Institutional Review Board for Human Subjects Research of Xi'an No. 4 Hospital (reference number: 20190012). Peripheral blood samples were diluted in phosphate buffer saline (PBS; Sigma-Aldrich, USA, Cat# P5493), slowly added to Lymphoprep (MP biomedicals, USA, Cat# 0916922-CF), and then centrifuged. The separated PBMNCs were collected and washed twice with PBS. CD3^+^ T cells were then purified from PBMNCs using an anti-human CD3 antibody (BioLegend, USA, Cat# 300308) with an immunomagneto cell sorting system (Miltenyi Biotec, Germany). Purified CD3^+^ T cells at 5 × 10^5^ cells/mL were plated in RPMI 1640 complete medium (Gibco, USA, Cat# 11875093) and were stimulated by adding 2 μg/mL CD3 antibody (Novoprotein, China, Cat# GMP-A018) and 2 μg/mL CD28 antibody (Novoprotein, China, Cat# GMP-A063). Stimulated T cells were then co-cultured with 2D- or 3D-preconditioned MSCs at a proportion of 10:1 for 3 days.

### Colitis modeling and therapy

Autoimmune colitis was induced in mice by drinking water administration of 2.5% DSS (MP Biomedicals, USA, Cat# 02160110-CF) for 5 days followed by normal purified water feeding. Mice of the Ctrl group were fed with purified water. At Day 3, indicated mice were intravenously infused with 1 × 10^6^ MSCs dissolved in PBS, and equal volume of PBS was injected as the control. Body weight of mice was recorded daily, and DAI was daily calculated according to changes in body weight, diarrhea and hematochezia, as previously described 68. At Day 7, mice were sacrificed, and colon tissues and serum samples were collected for subsequent analyses.

### T1D modeling and therapy

The mouse model of T1D was induced by intraperitoneal injection of 50 mg/kg/d STZ (MP Biomedicals, USA, Cat# 02100557-CF) consecutively for 5 days 45. Established T1D mice were selected for following experiments with the levels of random blood glucose higher than 11.1 mmol/L. At Day 7 and Day 14, indicated mice were intravenously infused with 1 × 10^6^ MSCs dissolved in PBS, and equal volume of PBS was injected as the control. Mice were recorded for their random blood glucose levels during the experiment using an ACCU-CKEK glucometer (Roche, Germany) following tail vein-puncture of whole blood sampling. The concentrations of HbA1c were determined using the A1CNow Self Check system (Sinocare, China). At Day 21, mice were sacrificed, and pancreas tissues and serum samples were collected for subsequent analyses.

### Chemical treatments *in vitro*

PGE2 (Cat# HY-101952) and VP (Cat# HY-B0146) were purchased from MedChemExpress, China. MSCs pretreated by 10 μM PGE2 or VP as respectively a chemical YAP1 promoter 69 or inhibitor 70 were performed for 48 h at the 2D- or 3D-condition prior to subsequent experiments.

### Flow cytometric analyses

For surface markers of MSCs, approximately 5 × 10^5^ MSCs after 2D- or 3D-preconditioning were harvested. Single-cell suspensions were prepared and were incubated with the following fluorescence-conjugated antibodies: FITC-CD73 (BD Bioscience, USA, Cat# 561254), PE-CD90 (BD Bioscience, USA, Cat# 561970), PE-CD105 (BD Bioscience, USA, Cat# 560839), PE-HLA-DR (BD Bioscience, USA, Cat# 555561), PE-CD34 (BD Bioscience, USA, Cat# 550761), and APC-CD45 (BD Bioscience, USA, Cat# 555485). For T-cell subsets, T cells after co-culture with MSCs were stained with the following fluorescence-conjugated antibodies: PE-CD4 (BioLegend, USA, Cat# 357404), PerCP/Cy5.5-IFN-γ (Thermo Fisher Scientific, USA, Cat# 45-7319-41), APC-IL-4 (Thermo Fisher Scientific, USA, Cat# 17-7049-41), FITC-IL-17A (BioLegend, USA, Cat# 512303), and Foxp3 (Thermo Fisher Scientific, USA, Cat# 88-8999-40). The samples were measured by flow cytometric analysis using a Beckman Coulter Epics XL cytometer (Beckman Coulter, USA).

### Colony-forming analysis

2D- or 3D-preconditioned MSCs were harvested and 3 × 10^2^ cells were seeded on 6-well plates containing α-MEM medium with 10% FBS. The medium was refreshed every 3 days. After 10 days, the wells were rinsed with PBS, and cells were fixed with 4% paraformaldehyde (PFA; Sigma-Aldrich, USA, Cat# 158127). The colonies were stained by 1% (m/v) crystal violet solution (Solarbio, China, Cat# C8470) 71.

### Proliferation analysis

2D- or 3D-preconditioned MSCs were seeded on 96-well plates at 5 × 10^2^ cells/well. Cell proliferation was analyzed daily with the CCK8 kit (Yeasen Biotech, China, Cat# 40203ES60) according to the standard protocol. Briefly, 10 μL CCK8 solution per 100 μL complete medium was added into wells, and the plates were incubated in 37°C and 5% CO_2_ for 3 h. The optical density value was recorded by a microplate reader (Bio-TEK Instruments, USA) at 450 nm, and cell numbers were normalized according to optical density values.

### Cell senescence analysis

2D- or 3D-preconditioned MSCs were seeded on 12-well plates at 1 × 10^5^ cells/well. Cell senescence was analyzed with the SA-β-gal staining kit (Beyotime, China, Cat# C0602) according to the manufacturer's instructions 71. The percentage of SA-β-gal positive cells was determined using the ImageJ 1.47 software (NIH, USA).

### Histopathological tissue analysis

At sacrifice, mouse colon or pancreas tissues were fixed with 4% PFA overnight at 4°C. Tissues were then embedded in paraffin, sectioned and subjected to H&E staining. The conditions of inflammation and edema of colons were evaluated under a light microscopy 68. Examination of pancreatic islets was performed with quantification of islet area percentages 45.

### ELISA

For serum examination, before sacrifice, samples of the whole peripheral blood were collected from the retro-orbital venous plexus at 500 μL. For media examination, after T-cell co-culture with MSCs, samples of conditional media were collected at 200 μL. The serum and cell-free media were isolated by centrifuging at 3,000 rpm for 10 min followed by 12,000 rpm for 10 min at 4°C to remove cell debris 45. C-peptide (Fankewei, China, Cat# F2580-A) and TNF-α (Neobioscience, China, Cat# EMC102a) in serum, as well as IL-6 (Neobioscience, China, Cat# EHC007) and TGF-β1 (Neobioscience, China, Cat# EHC107b) in media, were detected using ELISA kits according to the manufacturers' instructions.

### RNA-seq analysis

Total RNA of MSCs was isolated using the TRIzol Reagent (Invitrogen, USA, Cat# 15596026). The RNA was sequenced on a MGISEQ-2000 system (MGITECH, China). DEGs were identified by Dr. Tom 2.0. Subsequent functional analysis was performed utilizing the KEGG database or through GSEA analysis.

### qRT-PCR analysis

Total RNA was isolated from MSCs using the TRIzol Reagent (Invitrogen, USA, Cat# 15596026). cDNA was reverse-transcribed using the PrimeScript RT reagent kit (TaKaRa, Japan, Cat# RR036A) with oligo-dT and random primers following the manufacturer's instruction 72. Gene expression was detected using the TB Green Premix Ex Taq II kit (TaKaRa, Japan, Cat# RR820L) and the CFX96 Real-time RT-PCR System (Bio-Rad, USA). *YAP1* and *TGF-β1* mRNA levels were quantified by the 2^-ΔΔCT^ method with* Glyceraldehyde-3-Phosphate Dehydrogenase* (*GAPDH*) as an internal reference. The following primer sequences were used: *YAP1* forward primer: 5′-ACGTTCATCTGGGACAGCAT-3′; *YAP1* reverse primer: 5′-GTTGGGAGATGGCAAAGACA-3′; *TGF-β1* forward primer: 5′-TTGACGTCACTGGAGTGTG-3′; *TGF-β1* reverse primer: 5′-CGTTGATGCCACTTGAAAGC-3′; *GADPH* forward primer: 5′-GCACCGTCAAGGCTGAGAAC-3′; and *GADPH* reverse primer: 5′-TGGTGAAGACGCCAGTGGA-3′).

### WB analysis

Whole lysates of MSCs were prepared using the RIPA Lysis Buffer (Beyotime, China, Cat# P0013B). Proteins were extracted and the protein concentration was quantified using the BCA method (Beyotime, China, Cat# P0010). Equal amounts of protein samples were loaded onto sodium dodecyl sulfate-polyacrylamide gel electrophoresis (SDS-PAGE) gels and transferred to polyvinylidene fluoride (PVDF) membranes (Millipore, USA, Cat# ISEQ00010) which were blocked with 5% bovine serum albumin (BSA) (Gemini Bio, USA, Cat# 700-100P) in Tris buffered saline (TBS) for 2 h at room temperature. Then, the membranes were incubated overnight at 4°C with the following primary antibodies: anti-YAP1 (Abcam, UK, Cat# ab56701; diluted at 1:1000) and anti-GAPDH (CWBio, China, Cat# CW0100; diluted at 1:1000). After washing with TBS containing 0.1% Tween-20, membranes were incubated with a horseradish peroxidase (HRP)-conjugated Goat Anti-Mouse secondary antibody (Signalway, China, Cat# L3032) for 1 h at room temperature. The protein bands were visualized using an enhanced chemiluminescence kit (Amersham Biosciences, USA) and detected by a gel imaging system (Tanon, China) 72, 73. Quantification of WB results was performed using the ImageJ 1.47 software (NIH, USA).

### IF staining

For tissues, fresh colon and pancreas tissue samples were fixed in 4% PFA at 4°C for 4 h, washed with PBS, and dehydrated with 30% sucrose for 24 h. After dehydration, the samples were embedded in an optimal cutting temperature (OCT) compound, and 10 μm cryosections were prepared. The air-dried cryosections were permeabilized by 0.3% Triton X-100 (Solarbio, China, Cat# T8200) for 20 min at room temperature, following by blocking in goat serum (Boster, China, Cat# AR0009) for 30 min at room temperature. The cryosections were then incubated with the following primary antibodies overnight at a concentration of 1:100 at 4°C: a rat anti-CD4 antibody (Absin, China, Cat# abs174032) with a rabbit anti-IFN-γ antibody (Absin, China, Cat# abs119966) or a rabbit anti-IL-17 antibody (Absin, China, Cat# abs121447). After washing with PBS, sections were then stained with the following fluorescence-conjugated secondary antibodies at a concentration of 1:200 at 4°C for 1 h at room temperature: an Alexa Fluor 594-conjugated Goat Anti-Rabbit secondary antibody (Yeasen Biotechnology, China, Cat# 33112ES60) and an Alexa Fluor 488 Donkey Anti-Rat secondary antibody (Yeasen Biotechnology, China, Cat# 34406ES60). Sections were counterstained with Hoechst 33342 (Yeasen Biotechnology, China, Cat# 40731ES10). For MSCs, cells seeded on coverslips were washed, fixed in 4% PFA for 30 min at room temperature, treated with 0.3% Triton X-100 (Solarbio, China, Cat# T8200) diluted in PBS for 20 min at room temperature, blocked with 10% goat serum (Boster, China, Cat# AR0009) for 1 h at room temperature, and stained with an anti-YAP1 primary antibody (Abcam, UK, Cat# ab56701; diluted at 1:100) and an anti-β-tubulin primary antibody (Abcam, UK, Cat# ab314069; diluted at 1:100) overnight at 4ºC. After washing with PBS, coverslips were stained with an Alexa Fluor 594 Goat Anti-Mouse secondary antibody (Yeasen Biotechnology, China, Cat# 33212ES60) or an Alexa Fluor 488 Donkey Anti-Rabbit secondary antibody (Yeasen Biotechnology, China, Cat# 34206ES60) for 1 h at room temperature at a concentration of 1:200, and counterstained with Hoechst 33342 (Yeasen Biotechnology, China, Cat# 40731ES10). Fluorescent images were obtained using a confocal laser scanning microscope (Olympus, Japan) 72, 74, 75.

### Statistical analysis

Data were presented as mean ± standard deviation (SD). Data were analyzed by two-tailed unpaired Student's *t* test for two-group comparisons, one-way analysis of variation (ANOVA) followed by the Bonferroni's post-hoc test for multiple comparisons using the Prism 5.01 software (GraphPad, USA). *P* values of less than 0.05 were considered statistically significant.

## Supplementary Material

Supplementary figure and table.

## Abbreviations

2D: two-dimensional
3D: three-dimensional
α-MEM: alpha-minimum essential medium
ANOVA: one-way analysis of variation
BSA: bovine serum albumin
CFU: colony formation units
Ctrl: control
DEGs: differentially expressed genes
DSS: dextran sulfate sodium
ELISA: enzyme-linked immunosorbent assay
FBS: fetal bovine serum
Foxp3: Forkhead box protein P3
GAPDH: glyceraldehyde-3-phosphate dehydrogenase
GSEA: gene set enrichment analysis
GVHD: graft-versus-host disease
HbA1c: glycated hemoglobin
HLA-DR: human leukocyte antigen DR
HRP: horseradish peroxidase
H&E: hematoxylin and eosin
IBD: inflammatory bowel disease
IF: immunofluorescence
IFN-γ: interferon gamma
IL-17: interleukin 17
IL-4: interleukin 4
IL-6: interleukin 6
KEGG: Kyoto Encyclopedia of Genes and Genomes
MSCs: mesenchymal stem cells/mesenchymal stromal cells
OCT: optimal cutting temperature
P15: the fifteenth passages
P5: the fifth passages
PBMNCs: peripheral blood mononucleated cells
PBS: phosphate buffer saline
PCA: principal component analysis
PFA: paraformaldehyde
PGE2: prostaglandin E2
PVDF: polyvinylidene fluoride
qRT-PCR: quantitative real-time polymerase chain reaction
RNA-seq: RNA sequencing
SA-β-gal: senescence-associated beta-galactosidase
SD: standard deviation
SDS-PAGE: sodium dodecyl sulfate-polyacrylamide gel electrophoresis
SLE: systemic lupus erythematosus
STZ: streptozotocin
T1D: type 1 diabetes
TAZ: transcriptional coactivator with a PDZ-binding domain
TBS: Tris buffered saline
TCP: tissue culture plastic
TGF-β1: transforming growth factor-beta1
Th1: T helper 1
Th17: T helper 17
Th2: T helper 2
TNF-α: tumor necrosis factor-alpha
Treg: T regulatory
VP: verteporfin
WB: Western blot
YAP1: Yes-associated protein 1
