# Supplementary material for: YAP1 mediates the dimensional and chemical coordination of immunoregulation and therapy in extensively passaged mesenchymal stem cells
Source: Theranostics. 2025 Jan 6;15(5):1930–48. doi: 10.7150/thno.103314 (PMC11780522; doi:10.7150/thno.103314)
Supplement: Supplementary file 1 — Supplementary figure and table. [file thnov15p1930s1.pdf]

**Supplementary Figure and Legend**

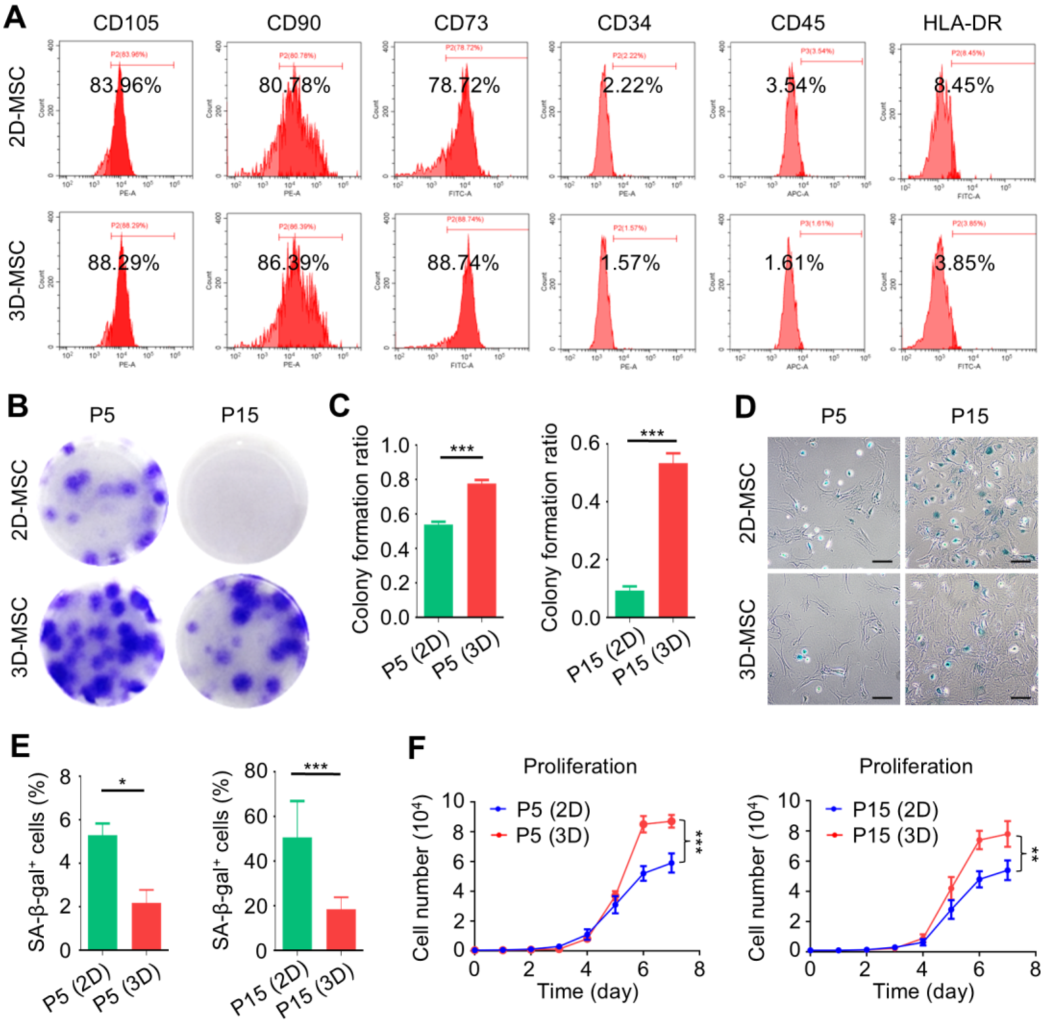


**Figure S1. Three-dimensional culture rescues replicative senescence of MSCs despite extensive passages.** (A) Flow cytometric analysis of surface markers of MSCs. (B) Colony formation of MSCs analyzed by crystal violet staining. (C) Quantification of colony forming units of MSCs over total seeded cells. (D) Cell senescence of MSCs analyzed by SA-β-gal staining. Scale bars = 100 μm. (E) Quantification of senescent percentages of MSCs. (F) Proliferation rate of MSCs analyzed by CCK8. $N = 3$ per group. Mean ± SD. *, $P < 0.05$; **, $P < 0.01$; ***, $P < 0.001$. Two-tailed unpaired Student's *t* test.

**Supplementary Table**

**Table S1. Key resources table.**

| REAGENT or RESOURCE | SOURCE | IDENTIFIER |
|---|---|---|
| Antibodies | | |
| FITC Mouse Anti-Human CD73 | BD Bioscience | Cat# 561254, RRID: AB_10894209 |
| PE Mouse anti-Human CD105 | BD Bioscience | Cat# 560839, RRID: AB_2033932 |
| PE Mouse Anti-Human CD90 | BD Bioscience | Cat# 561970, RRID: AB_395970 |
| PE Mouse Anti-Human HLA-DR | BD Bioscience | Cat# 555561, RRID: AB_395943 |
| PE Mouse Anti-Human CD34 | BD Bioscience | Cat# 550761, RRID: AB_393871 |
| APC Mouse Anti-Human CD45 | BD Bioscience | Cat# 555485, RRID: AB_398600 |
| PE anti-human CD3 Antibody | BioLegend | Cat# 300308, RRID: AB_314044 |
| Recombinant anti-Human CD3 mAb | Novoprotein | Cat# GMP-A018, RRID: N/A |
| Recombinant anti-Human CD28 mAb | Novoprotein | Cat# GMP-A063, RRID: N/A |
| PE anti-human CD4 Antibody | BioLegend | Cat# 357404, RRID: AB_312696 |
| IFN gamma Monoclonal Antibody (4S.B3), PerCP-Cyanine5.5 | Thermo Fisher Scientific | Cat# 45-7319-41, RRID: AB_10718246 |
| IL-4 Monoclonal Antibody (8D4-8), APC | Thermo Fisher Scientific | Cat# 17-7049-41, RRID: AB_10853508 |
| FITC anti-human IL-17A Antibody | BioLegend | Cat# 512303, RRID: AB_961391 |
| Rat anti-CD4 Monoclonal Antibody | Absin | Cat# abs174032 |
| Rabbit anti-IFN-γ Polyclonal Antibody | Absin | Cat# abs119966 |
| Rabbit anti-IL-17 Polyclonal Antibody | Absin | Cat# abs121447 |
| Anti-YAP1 Antibody | Abcam | Cat# ab56701, RRID: AB_2219140 |
| Alexa Fluor 488 Donkey Anti-Rabbit lgG(H+L) | Yeasen Biotechnology | Cat# 34206ES60, RRID: N/A |
| Alexa Fluor 488 Donkey Anti-Rat lgG(H+L) | Yeasen Biotechnology | Cat# 34406ES60, RRID: N/A |

| | | |
|---|---|---|
| Alexa Fluor 594 Goat Anti-Rabbit lgG(H+L) | Yeasen Biotechnology | Cat# 33112ES60, RRID: N/A |
| Alexa Fluor 594 Goat Anti-Mouse lgG(H+L) | Yeasen Biotechnology | Cat# 33212ES60, RRID: N/A |
| Goat Anti-Mouse IgG Antibody, HRP-conjugated | Signalway | Cat# L3032, RRID: AB_895481 |
| Anti GAPDH Mouse Monoclonal Antibody | CWBio | Cat# CW0100, RRID: AB_2801390 |
| Anti β-Tubulin Antibody | Abcam | Cat# ab314069, RRID: N/A |
| Chemicals, Peptides, and Recombinant Proteins | | |
| Hoechst 33342 | Yeasen Biotechnology | Cat# 40731ES10 |
| Trypsin | Gibco | Cat# 15050057 |
| Lymphoprep | MP biomedicals | Cat# 0916922-CF |
| Prostaglandin E2 | MedChemExpress | Cat# HY-101952 |
| Verteporfin | MedChemExpress | Cat# HY-B0146 |
| Red Blood Cell Lysis Buffer | Solarbio | Cat# R1010 |
| Dextran sulfate sodium salt | MP Biomedicals | Cat# 02160110-CF |
| Paraformaldehyde | Sigma-Aldrich | Cat# 158127 |
| Streptozotocin | MP Biomedicals | Cat# 02100557-CF |
| Crystal violet | Solarbio | Cat# C8470 |
| Triton X-100 | Solarbio | Cat# T8200 |
| Methanol | Kemao | N/A |
| Ethanol | Fuyu | N/A |
| Goat serum | Boster | Cat# AR0009 |
| Matrigel | Corning | Cat# 356231 |
| Dimethyl sulfoxide | Solarbio | Cat# D8370 |
| TRIzol | Invitrogen | Cat# 15596026 |
| Tris (Hydroxymethyl) Aminomethane | Solarbio | Cat# T8060 |
| Sodium dodecylsulfate | Solarbio | Cat# S8010 |
| Glycine | Solarbio | Cat# G8200 |
| Bovine serum albumin | Gemini Bio | Cat# 700-100P |
| 20x TBS buffer | Coolaber | Cat# SL1327 |
| SWE Fast High Resolution Electrophoresis Buffer (Dry Powder) | Servicebio | Cat# G2081 |
| PVDF, 0.2 µm, 26.5 cm x 3.75 m | Millipore | Cat# ISEQ00010 |
| Critical Commercial Assays | | |
| Cell counting kit-8 | Yeasen Biotech | Cat# 40203ES60 |
| Human Regulatory T Cell Staining Kit | Thermo Fisher Scientific | Cat# 88-8999-40 |
| Human TGF-β1 ELISA kit | Neobioscience | Cat# EHC107b |
| Human IL-6 ELISA kit | Neobioscience | Cat# EHC007 |
| Mouse TNF-α ELISA kit | Neobioscience | Cat# EMC102a |
| Mouse C-peptide ELISA Kit | Fankewei | Cat# F2580-A |

| | | |
|---|---|---|
| BCA Protein Quantitative Kit | Beyotime | Cat# P0010 |
| Cell Total RNA isolation Kit | Foergene | Cat# RE03113 |
| PrimeScript$^{TM}$ RT reagent Kit | Takara | Cat# RR036A |
| TB Green® Premix Ex Taq II kit | Takara | Cat# RR820L |
| Hematoxylin and Eosin Staining Kit | Beyotime | Cat# C0105M |
| Senescence β-Galactosidase Staining Kit | Beyotime | Cat# C0602 |
| RIPA buffer (high) | Beyotime | Cat# P0013B |
| Enhanced Chemiluminescence Kit | Amersham Biosciences | N/A |
| PAGE Gel Fast Preparation Kit | Epizyme Biotech | Cat# PG112 |
| Medium | | |
| α-MEM | Gibco | Cat# 12000063 |
| Cell recovery solution | Corning | Cat# 354253 |
| FBS | Gibco | Cat# 16140071 |
| RPMI 1640 | Gibco | Cat# 11875093 |
| Penicillin-Streptomycin | Gibco | Cat# 115140122 |
| PBS | Sigma-Aldrich | Cat# P5493 |
| Experimental Models: Cell Lines | | |
| N/A | | |
| Experimental Models: Organisms/Strains | | |
| Mouse: C57BL/6J | Laboratory Animal Center of the Fourth Military Medical University | N/A |
| Oligonucleotides | | |
| qRT-PCR primers | Sangon Biotech | N/A |
| Recombinant DNA | | |
| N/A | | |
| Software and Algorithms | | |
| Gen5 | Bio-Tek | RRID: SCR_017317 |
| GraphPad Prism | GraphPad | RRID: SCR_002798 |
| ImageJ 1.47 | National Institute of Health | RRID: SCR_003070 |
| CytExpert Software2.4 | Beckman Coulter | RRID: SCR_017217 |
| Bio-Rad CFX Maestro | Bio-Rad | N/A |
| Adobe Photoshop | Adobe Systems Software Ireland Ltd | N/A |
| Microsoft Office | Microsoft | N/A |
| OLYMPUS FLUOVIEW FV31S-SW | OLYMPUS | N/A |
| OlyVIA/XV Imaging | Olympus Soft Imaging Solutions GmbH | N/A |
| BGISEQ-500 | https://www.bgi.com/global/company/careers/bgi-launches-its-desktop-sequencer-bgiseq-500/ | RRID:SCR_017979 |

| | | |
|---|---|---|
| DESeq2 | https://bioconductor.org/packages/release/bioc/html/DESeq2.html | RRID: SCR_015687 |
| R (4.3.0) | https://www.r-project.org/ | RRID:SCR_001905 |
